# FTY720 attenuates excitotoxicity and neuroinflammation

**DOI:** 10.1186/s12974-015-0308-6

**Published:** 2015-05-08

**Authors:** Raffaela Cipriani, Juan Carlos Chara, Alfredo Rodríguez-Antigüedad, Carlos Matute

**Affiliations:** Centro de Investigaciones Biomédicas en Red (CIBERNED), Achucarro Basque Center for Neuroscience Bizkaia Science and Technology Park, Building 205, E-48170 Zamudio, Spain; Departamento de Neurociencias, Universidad del País Vasco, Barrio Sarriena s/n, E-48940 Leioa, Spain; Servicio de Neurología, Hospital de Basurto, C/Avda. de Montevideo 18, 48013 Bilbao, Spain

**Keywords:** FTY720, Neurons, NMDA, Kainic acid, Neuroinflammation, Microglia, LPS

## Abstract

**Background:**

FTY720 (fingolimod, Gilenya™), a structural analog of sphingosine-1-phosphate (S1P), is the first oral drug approved for treatment the relapsing-remitting form of multiple sclerosis (MS), and its efficacy has been related to induced lymphopenia and consequent immunosuppression via modulation of S1P_1_ receptors (S1P_1_R). However, due to its lipophilic nature, FTY720 crosses the blood brain barrier (BBB) and could act directly on neural cells. In this study, we investigated the effectiveness of FTY720 as a neuroprotective agent using *in vitro* and *in vivo* models of excitotoxic neuronal death and examined if FTY720 exerts a direct action on neurons, or/and an indirect modulation of inflammation-mediated neurodegeneration as a possible mechanism of neuroprotection.

**Methods:**

Primary neuronal and organotypic cortical cultures were treated with N-methyl-D-aspartic acid (NMDA) to induce excitotoxic cell death (measured by lactate dehydrogenase (LDH) assay or propidium iodide uptake, respectively). The effects of FTY720 treatment (10, 100 and 1,000 nM) on neuronal survival were examined. As an *in vivo* model of neuronal death and inflammation, we used intracerebroventricular (*icv*) administration of kainic acid (KA; 0.5 μg/2 μl) in Sprague–Dawley rats. FTY720 was applied *icv* (1 μg/2 μl), together with KA, plus intraperitoneally (*ip*; 1 mg/kg) 24 h before, and daily, until sacrifice 3 days after *icv*. Rats were evaluated for neurological score, neuronal loss in CA3 hippocampal region and activation of microglia at the lesion site. In addition, we tested FTY720 as a modulator of microglia responses using microglial cell cultures activated with lipopolysaccharide (LPS) and its effects in stress signalling pathways using western blotting for p38 and JNK1/2 mitogen-activated protein kinases (MAPKs).

**Results:**

FTY720 was able to reduce excitotoxic neuronal death *in vitro*. Moreover, *in vivo* repeated FTY720 administration attenuated KA-induced neurodegeneration and microgliosis at the CA3 lesion site. Furthermore, FTY720 negatively modulates p38 MAPK in LPS-activated microglia, whereas it had no effect on JNK1/2 activation.

**Conclusions:**

These data support a role for FTY720 as a neuroprotective agent against excitotoxin-induced neuronal death and as a negative modulator of neuroinflammation by targeting the p38 MAPK stress signalling pathway in microglia.

**Electronic supplementary material:**

The online version of this article (doi:10.1186/s12974-015-0308-6) contains supplementary material, which is available to authorized users.

## Background

FTY720 (fingolimod, Gilenya™) is an immunomodulatory drug approved in 2010 as the first oral treatment for relapsing-remitting multiple sclerosis (MS) [1,2]. The bio-active form, FTY720-phosphate (FTY720-P), derived from the phosphorylation by sphingosine kinase 2 (SphK2), is a structural analogous of Sphingosine-1-phosphate (S1P), and it targets all S1P receptors (S1P_1–5_) with the exception of S1P_2_ [3,4]. The main and well-characterized action of FTY720 is the sequestration of circulating mature lymphocytes to secondary lymphoid organs via modulation of S1P_1_ receptors [4,5]. The consequent important immunosuppressive function gave positive results in rodent models of MS like experimental autoimmune encephalomyelitis (EAE) [3,6] and underlies its therapeutic activity in MS. However, contributing mechanisms other than influence on lymphocytes trafficking have been reported for FTY720’s effects in EAE [7-9] and in alternative MS models that exclude the immunological compartment [10,11]. Moreover, the high lipophilic FTY720 crosses blood brain barrier (BBB) and accumulates in the brain and spinal cord [12], and endogenous SphK2 [13] and all the S1P receptors, with the exception of S1P_4_, are abundantly expressed in the brain [14], further suggesting that action of FTY720 might involve direct effects in the central nervous system (CNS) and implying a therapeutic potential of FTY720 also in other neuropathologies.

FTY720 has a potent anti-inflammatory effect in animal models of CNS injury, such as brain ischemia [15-17], intracerebral haemorrhage [18,19], spinal cord injury [20], Alzheimer’s disease [21-24], glioblastoma [25] and epilepsy [26]. *In vitro* studies confirm direct action of FTY720 on microglial cells, reducing the production of pro-inflammatory cytokines from LPS-stimulated microglia [27] or in a model of transient demyelination in rat CNS reaggregate spheroid cell culture [28]. Notably, FTY720 also enhanced the production of neurotrophic factors, brain-derived nerve factor (BDNF) and glial-derived nerve factor (GDNF) by LPS-activated microglia, [27], suggesting a FTY720-mediated shift towards a neuroprotective microglial phenotype. However, a direct action of FTY720 on neurons remains unclear. Recent studies demonstrate that, in cultured neurons, FTY720 counteracts N-methyl-D-aspartic acid (NMDA) or oligomeric amyloid β (Aβ)-induced neuronal death [21,29,30] increasing the neuronal production of BDNF [21,29].

A very useful tool to study neuronal death and neuroinflammation *in vivo* is the administration of kainic acid (KA) in rodents: this is a well-established model of excitotoxicity and seizure-related neurological diseases, since it induces seizures in animals, followed by an acute neurodegeneration confined in specific regions of the brain, the most vulnerable being the hippocampus [31,32]. KA-induced damage is accompanied by inflammatory responses, a very common feature of neurodegenerative diseases, driven initially by microglial cells and astrocytes, and producing nitric oxide and pro-inflammatory cytokines which ultimately exacerbate neuronal injury and contribute to the expansion of brain damage [33-35].

Given the not fully understood action of FTY720 in brain injury, in this study we investigated how this drug might confer neuroprotection after excitotoxicity *in vitro* and *in vivo*. We demonstrate that daily treatment with FTY720 results in a reduction of neuronal loss in CA3 regions of the hippocampus after intracerebroventricular (*icv*) injection of KA and in a pronounced decrease of activated microglia at the site of injury. In addition, our *in vitro* results indicate that FTY720 modulates inflammatory phenotype of LPS-activated microglial cells and protects neuronal cortical cultures from NMDA-excitotoxicity, further supporting a direct action of FTY720 as neuroprotective and anti-inflammatory agent in brain injury conditions.

## Methods

### Reagents and chemicals

FTY720 (2-amino-2-[2-(4-octylphenyl)ethyl]propane-1,3-diol) and FTY720-P (2-amino-2[2-(4-octylphenyl)ethyl]-1,3-propanediol, mono dihydrogen phosphate ester) were provided by Novartis. For *in vitro* experiments, powdered FTY720 and FTY720-P were reconstituted in dimethyl sulfoxide hydrochloric acid (DMSO)/50 mM HCl; for the intraperitoneal (*ip*) and *icv* injections, FTY720 was diluted in vehicle (saline solution). For the *ip* injection, FTY720 was freshly prepared every day. KA was from Abcam (Cambridge, USA); MK801 was from Tocris (Bristol, UK). NMDA, propidium iodide (PI) and LPS (*Escherichia coli* O11:B4) were from Sigma-Aldrich (St. Louis, USA). All cell culture supplies, if not otherwise indicated, were purchased from Gibco (Life Technologies, Madrid, Spain). Cytotoxicity 96 colorimetric assay for the quantification of lactate dehydrogenase (LDH) release was purchased from Promega Corporation (Madison, USA).

### Animals

All procedures and experiments involving animals and their care were carried out according to the guidelines of the European Union Council (Directive 2010/63/EU) and Spanish regulations (Real Decreto 53/2013) on animal ethics and welfare, and under the supervision and with the approval of our internal animal ethics committee (University of the Basque Country, UPV/EHU). All possible efforts were made to minimize animal suffering and the number of animals used.

### Neuronal primary cultures

Cortical neurons were obtained from the cortical lobes of E18 Sprague–Dawley rat embryos as previously described [36]. Briefly, neurons were resuspended in Neurobasal-B27 medium plus 10% fetal bovine serum and then seeded onto poly-L-ornithine (Sigma) coated 48-well plates at 1.5 × 10^5^ cells per well. The medium was replaced by serum-free Neurobasal-B27 medium 24 h later. The cultures were essentially free of astrocytes and microglia and were maintained at 37°C and 5% CO_2_. Cultures were used at 9 to 11 *days in vitro* (DIV).

### Organotypic cultures

Organotypic cerebrocortical cultures were obtained from brains of 7-day-old Sprague–Dawley rat pups using the method described by Plenz and Kitai [37] with minor modifications [38]. Briefly, brains were removed and the two hemispheres separated in Hank’s balanced salt solution (HBSS; Sigma-Aldrich), supplemented with Ca^2+^ and Mg^2+^. The thalamus and midbrain were discarded, and each hemisphere was sliced with a McIllwain tissue chopper (Mickle Laboratory Engineering Co., Surrey, UK) in order to obtain coronal slices of 400 μm of thickness. Slices containing cortex and striatum (but not hippocampus) were selected under a microscope and dissected to eliminate the corpus callosum. Slices were plated onto Millicell CM culture inserts (Millipore, Madrid, Spain) and maintained in 50% Neurobasal-B27, 25% inactivated horse serum, 25% HBSS, 5.5 mM glucose, 2 mM L-glutamine, Antibiotic-Antimycotic 1X (100 U/ml penicillin, 100 μg/ml streptomycin and 0.25 μg/ml amphotericin B), at 37°C and 5% CO_2_. Slices were used after 11 to 13 DIV.

### Microglial cells cultures

Rat primary microglia cultures were prepared from the cerebral cortex of newborn (P0-P2) Sprague–Dawley rats. Briefly, cortices were dissociated and plated with serum-supplemented Iscove’s modified Dulbecco’s medium. After 2 weeks, confluent monolayer of cultured astrocytes was depleted from microglia by mechanical shaking, according to standard protocols. Free-floating microglia were collected from shaken astrocyte flasks and purified by plating on non-coated plastic Petri dishes (Sterilin). After 48 h, non-adhered cells (progenitors cells) were eliminated and microglial cells were re-plated on poli-D-lysine (PDL; Sigma)-coated wells (1×10^5^ cells per well) in Dulbecco’s modified Eagle’s medium (DMEM) and 5% inactivated horse serum and used for the experiments 24 h after. The purity of cultured microglia was higher than 99% under these conditions.

### *In vitro* assays

#### Toxicity assays

Cortical neurons at 9 DIV were pre-treated for 24 h with FTY720 (10 to 1,000 nM) and then exposed to NMDA (25 and 100 μM) for additional 24 h in the same conditioned medium. Neuronal death was estimated by measuring the level of LDH released from damaged cells into the culture media. All experiments were performed at least in duplicate, and the values provided are the normalized mean ± SEM of at least four independent experiments.

For organotypic culture toxicity experiments, slices (11 DIV) were treated with NMDA 50 μM in HBSS (free of Ca^2+^ and Mg^2+^) containing 2.6 mM CaCl_2_, 4.5 mM glucose and 10 μM glycine for 30 min at 37°C, in the presence or in the absence of FTY720 (10 and 100 nM) or MK801 (50 μM, 10 min pre-incubation), as indicated. In addition, FTY720, when applied, was added 24 h before and 24 h after the treatment with NMDA. Twenty-four hours after stimulation with NMDA, the damaged region within the slice was determined by PI uptake, whose signal correlates with LDH release in models of excitotoxicity [39]. Briefly, slices were incubated in PI-containing medium (10 μM) for 1 h at 37°C and then washed out three times with culture medium. Fluorescence from dead cells, which take up the dye in the nucleus, was visualized and photographed using a fluorescence microscope Nikon AZ100. The captured images were analysed by ImageJ software (http://rsbweb.nih.gov/ij/download.html) to measure fluorescence intensity, representing PI uptake. For each image, the corrected total fluorescence (CTF) in the cerebral cortex was calculated in relation to the total cortical area in each slice using the formula:

$$ \mathrm{C}\mathrm{T}\mathrm{F}\kern0.5em =\kern0.5em  Integrated\  density\kern0.5em -\kern0.5em \left( Selected\  area\kern0.5em \ast \kern0.5em  Mean\  fluorescence\  of\  background\  readings\right) $$

Mean fluorescence of background was recorded from randomly selected square areas outside of the area of interest. Data, expressed as normalized mean ± SEM, were averaged from two similarly treated slices and at least four independent experiments run for each condition.

For the *in vitro* assays, the concentrations range (10 to 100 to 1,000 nM) was chosen in agreement with previous studies demonstrating efficacy of FTY720 and FTY720-P at nanomolar levels [14,29].

### Western blotting

For Western blotting assays, microglia cells on PDL-coated wells were stimulated in DMEM serum-free medium with LPS 100 ng/ml for the indicated time (time-course); in another set of experiments, microglia were pre-treated with FTY720 or FTY720-P (100 nM) for 24 h, and later stimulated in DMEM serum-free medium with LPS 100 ng/ml for 60 min, in the presence or in the absence of FTY720. After washing with ice-cold phosphate buffered saline (PBS), total protein was extracted from microglia cell cultures by scraping the cells in SDS/sample buffer. Samples were boiled for 10 min, separated by electrophoresis using Criterion TGX Precast 12% gels and transferred to Trans-Blot Turbo Midi PVDF Transfer Packs (Bio-Rad, Hercules, USA). For immunoblotting, membranes were blocked in 5% skimmed milk, 5% serum in tris-buffered saline/0.05% Tween-20 (TBS-T) and proteins detected by specific primary antibodies in 5% bovine serum albumin (BSA) in TBS-T overnight at + 4°C (all from Cell Signalling, Beverly, USA): phospho-p38 mitogen-activated protein kinase (MAPK) (#9211; 1:1,000); Phospho-SAPK/JNK (#9251; 1:1,000); total p38 MAPK (#9212; 1:1,000); total SAPK/JNK (#9252; 1:2,000). After washing, the membranes were incubated with horseradish peroxidase-conjugated secondary antibodies (1:2,000, Sigma) in 5% skimmed milk and 1% normal serum in TBS-T for 1 h at room temperature and developed using enhanced chemiluminiscence according to the manufacturer’s instructions (Super Signal West Dura or Femto, Pierce, Life Technologies, Madrid, Spain). Images were acquired with a ChemiDoc MP system (Bio-Rad) and quantified using Image Lab Software; values of phosphorylated protein were normalized to corresponding total protein signal and provided as the mean ± SEM of at least three independent experiments.

### *In vivo* model of neurodegeneration

We used a total of 37 adult male Sprague–Dawley rats (200 to 250 g), divided in six independent sets of experiments. Rats were kept on a 12/12 h light/dark cycle with constant ambient temperature and humidity. Food and water were available *ad libitum*. We used unilateral *icv* injection of KA as a model of neurodegeneration. Rats (*n* ≥ 3 per experimental group) were anesthetized by *ip* of ketamine 80 mg/kg (Imalgene®, Merial Laboratorios Sa, Barcelona, Spain) and xylazine 10 mg/kg (Rompun®, Bayer Hispania, S.L., Barcelona, Spain ) and placed into a stereotaxic apparatus (David Kopf Instruments, Los Angeles, USA). KA (0.5 μg in 2 μl in saline), alone or in combination with FTY720 (1 μg in 2 μl in saline) was injected into the right lateral ventricle (right side referred thereafter as ipsilateral), at the following coordinates from bregma: −1 mm anterioposterior, 2 mm mediolateral, 4 mm dorsoventral [40]. Injections were carried out over a 5-min period using an infusion pump (KD Scientific, Holliston, USA), with a constant infusion rate of 0.4 μl/min. Initially, we tested a single dose of FTY720 *icv* co-injected with KA. In a second set of experiments, we injected FTY720 together with KA, both *icv*, plus a prophylactic FTY720 injection (1 mg/kg; *ip*) 24 h before, and subsequent daily *ip* injections of vehicle or FTY720 until sacrifice 3 days after KA application. Control animals received vehicle only. The dose of 1 mg/kg was previously reported as effective in reducing neuroinflammation in rodent ischemic models [15,16] and in lithium-pilocarpine-induced status epilepticus [26]. Following surgery, three animals were housed per cage and observed for spontaneous seizure activity every 30 min for a total time of 120 min. Seizures were quantitatively scored as previously described, according to a modified Racine scale [41]: 0, normal; 1, immobilization; 2, forelimb and/or tail extension, rigid posture; 3, repetitive movements, head bobbing and gnawing; 4, rearing and falling; 5, continuous rearing and falling; 6, severe tonic-clonic seizure with loss of postural control and 7, death in the first 2 h. Seizure scoring was carried out blinded to the treatment received by the animals.

### Histology

After 72 h of *icv* injection, the animals were deeply anesthetized with chloral hydrate 400 mg/kg (Panreac Quimica, Barcelona, Spain) and transcardially perfused with 4% paraformaldehyde in 0.1 M PBS (pH 7.4). The brains were removed and immediately post-fixed in the same solution for 3 h. Then, they were washed and stored at + 4°C in PBS/Azide (0.02%) until sectioning. For all brains, six series of 40-μm-thick coronal sections at the level of the hippocampus were cut on a vibratome (HM 650 V, Microm International, Walldorf, Germany). One of the series was used for Nissl stain (toluidine blue, Sigma-Aldrich) to evaluate the lesion extent in the hippocampus; another series was processed with Fluoro-Jade C (FJC, Millipore), a specific marker for degenerating neurons [42]; the remaining sections were used for immunohistochemistry, as described below.

#### Quantification of the lesion size

Serial sections were stained with 1% toluidine blue solution (Nissl’s stain) to quantify lesion area in the hippocampus. A total of eight slices at the level of dorsal hippocampus was used per animal. The edge of the lesion, restricted to the CA3 region of the hippocampus, was determined by the border between the healthy and the pyknotic neurons. Images of stained ipsilateral hippocampus were acquired using Zeiss Axioplan 2 microscope coupled to an Axiocam MRc5 digital camera (Zeiss, Oberkochen, Germany) under a 4× objective. Microsoft Image Composite Editor Software (Microsoft Research) was used to reconstruct the entire hippocampus; then CA3 and hippocampal areas (mm^2^), referring to Paxinos and Watson rat atlas [40], and lesion area were measured for each slice using ImageJ software. Mean lesion area per animal was calculated and normalized to hippocampus mean area or CA3 mean area. Similarly, adjacent set of sections were used for FJC staining to detect dying neurons, as previously described [42]. Briefly, floating sections were mounted on gelatin-coated slides and dried at room temperature overnight. Sections were then rehydrated by sequential soaking in 100% ethanol (3 min), 70% ethanol (3 min) and distilled water (two times for 2 min). After 15 min incubation in 0.06% potassium permanganate, sections were rinsed for 1 min in distilled water and immersed in a solution containing 0.0001% solution of FJC dissolved in 0.1% acetic acid vehicle for 20 min. The slides were then rinsed through distilled water (three times for 1 min) and air dried room temperature overnight. The air dried slides were cleared in xylene (two times for 3 min) and then coverslipped with DPX non-fluorescent mounting media (Sigma). Sections were examined with a Zeiss Axioplan 2 microscope and images acquired using an Axiocam MRc5 digital camera (Zeiss).

#### Immunohistochemistry

Immunoperoxidase staining on free-floating sections was used for identification of neurons and microglial cells. Briefly, after quenching of endogenous peroxidase (H_2_O_2_ 0.3%) and blocking nonspecific binding with 4% normal goat serum, 0.1% Triton X-100 in PBS, we used the following primary antibodies: anti-neuronal nuclei (NeuN) (#ABN78, Millipore; 1:1,000); anti-ionized calcium-binding adapter molecule1 (Iba1) (#019-19741, Wako; 1:1,000). Subsequently, primary antibodies were detected using appropriated biotinylated secondary antibodies (1:200; Vector Laboratories, Burlingame, USA), followed by incubation with avidin-biotin-peroxidase complex (Vector Laboratories). Peroxidase activity was visualized by incubation in 3,3′-diaminobenzidine (DAB) substrate (Roche, Basel, Switzerland). Finally, the sections were mounted in gelatin-coated slides, dehydrated through graded alcohols, cleared with xylene and coverslipped with DPX. The sections were visualized using Zeiss Axioplan2 microscope coupled to an Axiocam MRc5 digital camera (Zeiss) to examine neuronal integrity and the extent of microgliosis, and representative photomicrographs of ipsilateral CA3 regions were taken under a 10× magnification objective. The extent of microgliosis was quantified by counting the number of Iba1^+^ cells in the ipsilateral CA3 hippocampus in three independent sections per animal, using three 40×−fields per section. Negative controls in all experiments included the omission of one of the primary antibodies and provided no labelling, indicating the reliability and specificity of the immunostaining.

### Statistical analysis

Data are expressed as mean ± SEM. Statistical analyses were performed using Prism version 4.0 (GraphPad Software, USA). Comparisons between two groups were analysed using unpaired,two-tailed Student’s *t* test or Mann–Whitney non-parametric test. Comparisons among multiple groups were analysed by one-way or two-way analysis of variance (ANOVA), as appropriate, followed by Bonferroni *post hoc* test. *P* ≤ 0.05 or *P* ≤ 0.01 was defined as significant or highly significant, respectively.

## Results

### FTY720 reduces excitotoxic neuronal death *in vitro*

**Figure 1:**
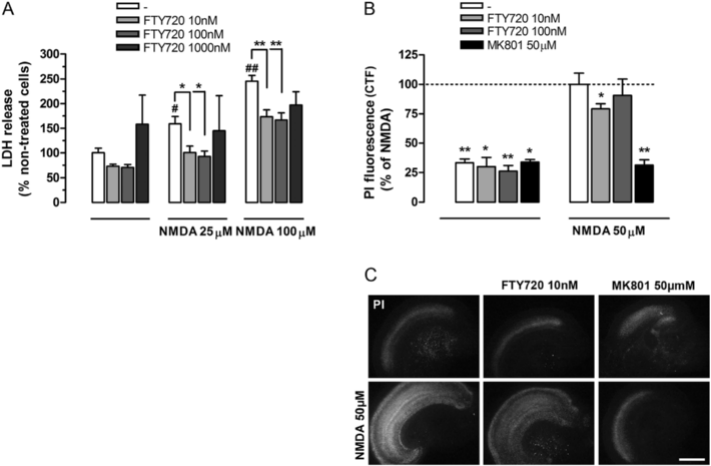
FTY720 prevents excitotoxic neuronal death in cerebrocortical primary and organotypic cultures. **(A)** Neuronal primary cultures 9 DIV were pre-treated with increased concentration of FTY720 (10 to 1,000 nM) for 24 h and then stimulated with N-methyl-D-aspartic acid (NMDA) (25 to 100 μM) in the same conditioned medium. After 24 h of NMDA treatment, lactate dehydrogenase (LDH) released in the medium was measured and mean ± SEM were reported as per cent of non-treated neurons (*n* = 6, except for FTY720 1 μM, *n* = 2; two-way ANOVA: * or ^#^ *P* < 0.05; ** or ^##^ *P* < 0.01; ^#^ *vs* control). **(B)** Organotypic cultures were pre-treated 24 h with FTY720 (10 and 100 nM) and then incubated with NMDA (50 μM) for 30 min, in the presence or in the absence of FTY720 or MK801 (50 μM, 10 min pre-incubation). FTY720 was added for additional 24 h. The damage was detected by PI uptake 24 h after treatment with NMDA and propidium iodide (PI) intensity was measured as corrected total fluorescence (CTF). Data are expressed as the mean ± SEM (*n* ≥ 4 independent experiments; in each experiment, at least two slices for condition were used) and referred to percent of NMDA-treated neurons (dotted line). Student’s *t* test: *P < 0.05 and **P < 0.01 *vs* NMDA-treated cells. **(C)** Representative photographs showing PI uptake in cortical slices treated as in (B). Scale bar: 1 mm.

For the induction of excitotoxic neuronal death, we used primary neuronal cultures and organotypic cortical cultures, two experimental settings that exclude confounding interactions of the peripheral immune system. Initial experiments with FTY720 alone showed that it was somewhat toxic, albeit not significant, at the highest concentration used (1,000 nM), an effect which could be due to receptor-independent mechanisms [43]. Subsequently, neuronal cultures were subjected to excitotoxic concentration of NMDA (25 and 100 μM), and neuronal death was assessed 24 h later measuring LDH released from damaged neurons in the medium. NMDA caused neuronal death in a concentration-dependent fashion. FTY720 (10 to 1,000 nM), applied to the cultures 24 h before NMDA and maintained in the medium thereafter, reduced LDH release and, therefore, neuronal damage, with higher dose being less effective if not slightly toxic (Figure 1A). This protective effect was also maintained at the higher concentration of NMDA.

To further assess the neuroprotective action of FTY720 against NMDA-excitotoxicity, we used cerebrocortical organotypic slices, a more integral *ex vivo* preparation that maintains many aspects of *in vivo* biology, including preserved brain architecture and cellular components representation. Moreover, organotypic cortical slices are vulnerable to NMDA receptor activation [36]. Cortical slices were pre-treated with FTY720 (10 and 100 nM) for 24 h and then incubated with NMDA (50 μM) for 30 min. Organotypic cultures were then washed out and maintained in culture medium for additional 24 h, in the presence or in the absence of FTY720, as indicated (Figure 1B). PI uptake was used to quantify NMDA-induced cell death, and fluorescence intensity was expressed as CTF referred to NMDA treatment as 100% (Figure 1B). NMDA treatment significantly increased the PI uptake as compared to control, an effect that was reduced by low concentrations of FTY720 (Figure 1B, C), in line with the results obtained in primary cultures. The NMDA receptor antagonist MK-801 was fully protective (Figure 1B).

### Prolonged treatment with FTY720 reduces hippocampal degeneration caused by *icv* injection of KA

**Figure 2:**
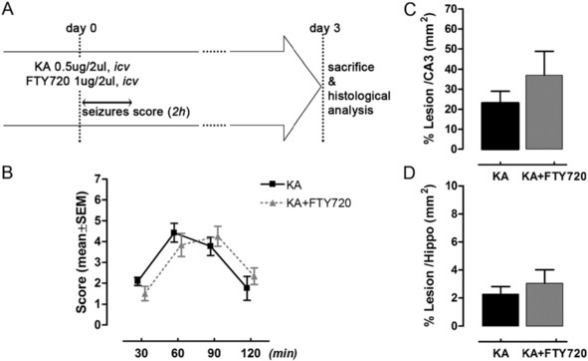
Single acute treatment with FTY720 does not protect from kainic acid (KA)-induced neurodegeneration *in vivo*. **(A)** Schematic timeline of experimental protocol. FTY720 was applied as a single *icv* dose together with KA. Seizure score was evaluated at the end of the surgery observing the animals during 30 min intervals over a period of 2 h. Animals were sacrificed 3 days after *icv* injection and brain tissues processed for immunohistochemical analysis. **(B)** Effect of FTY720 (1 μg/2 μl, *icv*) on seizure-like behaviour induced by KA *icv* injection (0.5 μg/2 μl). Seizure was scored as described in the ‘Methods’ section, with the higher score indicating greater seizure severity. The mean seizure score for KA group (*n* = 6) and KA + FTY720 group (*n* = 6) was plotted against time after *icv*. **(C, D)** Quantification of the extent of the lesion in animal *icv* injected with KA (*n* = 6) and KA + FTY720 (*n* = 6) after Nissl’s staining. Eight slices per animal were evaluated. Area of the lesion and areas of CA3 and hippocampus were measured for each slice (mm^2^) in the ipsilateral side. The mean lesion area per animal was calculated and normalized to the mean CA3 or hippocampal areas (C, D). Data are expressed as the mean ± SEM of *n* = 6 animals for each experimental group (two independent experiments with 3 animals in each group). There is no significant difference between the two groups (B: Mann–Whitney test at any time point of recorded seizure score; C and D: Student’s *t* test).

**Figure 3:**
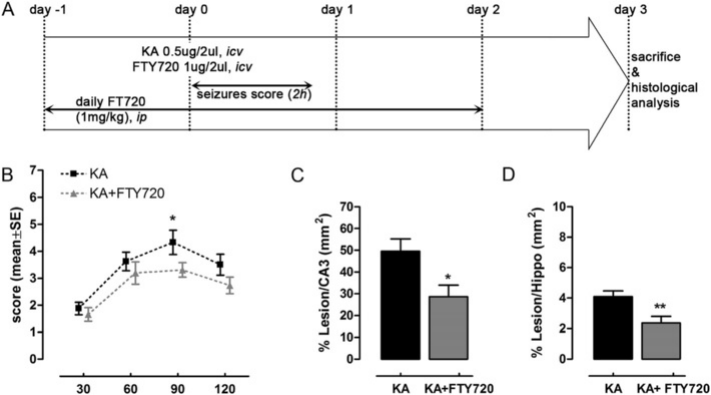
Repeated (daily) treatment with FTY720 protects from kainic acid (KA)-induced neurodegeneration *in vivo*. **(A)** Schematic timeline of experimental protocol. FTY720 or vehicle was *ip* injected (1 mg/kg) daily, starting 1 day before *icv* surgery up to 2 days thereafter. Animals received a single *icv* injection of KA alone (0.5 μg/2 μl) or in combination with FTY720 (1 μg/2 μl). Seizure score was evaluated post-surgery at 30-min intervals over a period of 2 h. On day 3 after *icv* injection, animals were sacrificed and brain tissues processed for immunohistochemical analysis. **(B)** Effect of daily FTY720 treatment on seizure-like behaviour induced by KA *icv* injection. Seizure was scored as described in the ‘Methods’ section, with the higher score indicating greater seizure severity. The mean seizure score for KA group (*n* = 12, four independent experiments with 3 animals each) and KA + FTY720 group (*n* = 13, four independent experiments with ≥3 animals each) was plotted against time after *icv*. *P < 0.05 *vs* KA-treated group, Mann–Whitney test at any time point of recorded seizure score. **(C, D)** Quantification of the extent of the lesion in animal *icv* injected with KA (*n* = 11) and KA + FTY720 (*n* = 12) after Nissl’s staining. Eight slices per animal were evaluated. Area of the lesion and areas of CA3 and hippocampus were measured for each slice (mm^2^) in the ipsilateral side. The mean lesion area per animal was calculated and normalized to the mean CA3 or hippocampal areas (C, D). Only ipsilateral hemisphere was considered for the analysis. *P < 0.05 and **P < 0.01 *vs* KA-treated group, Student’s *t* test. All data are expressed as the mean ± SEM of *n* ≥ 11 animals for each experimental group (four independent experiments).

**Figure 4:**
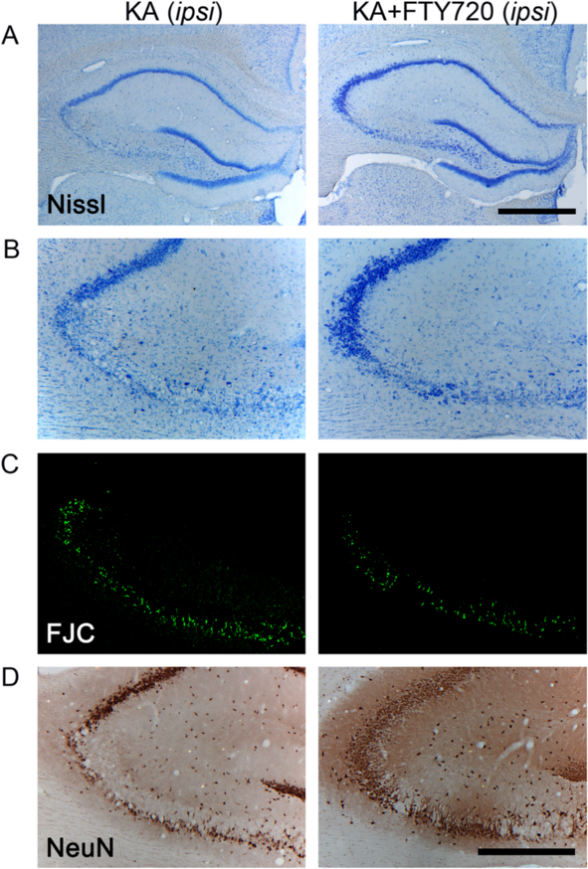
Histological analysis of the protective effect of FTY720 in CA3 region following *icv* injection of kainic acid (KA). Slices collected 3 days after *icv* surgery were stained with Nissl **(A, B)**, Fluoro-Jade C (FJC) **(C)**, and anti-NeuN antibody **(D)**. Figure shows representative photomicrographs of bright field (A, B, D) and epifluorescence (C) microscopy images obtained from coronal sections of KA-treated (left panel) and KA + FTY720-treated animal groups (right panel). Only the damaged ipsilateral hemisphere is shown. KA-induced cell death is evident in the CA3 region, as indicated by Nissl’s staining (A, B), lower number of NeuN^+^ cells (D), and increased density of FJC^+^ neurons (C). KA + FTY720-treated group (right panel) shows significant attenuated loss of neurons as compared to KA-treated group (left panel). (A) Dorsal ipsilateral hippocampus. Scale bar: 1 mm. (B-D) CA3 region of ipsilateral hippocampus. Scale bar 500 μm.

The results obtained *in vitro* attest the neuroprotective capacity of FTY720 against excitotoxic neuronal death. To further evaluate this feature, we tested FTY720 effects *in vivo*, in a model of neurodegeneration in rats using *icv* injection of KA. This potent excitotoxin induces cell death of selected populations of neurons in the hippocampus of treated animals, the most relevant being CA3 region. KA (0.5 μg/2 μl) was injected in the right lateral ventricle, and neuronal damage was evaluated 3 days later using Nissl’s and FJC staining, and immunohistochemistry with anti-NeuN antibody. In a first set of experiments, we applied a single dose of FTY720, *icv* (1 μg/2 μl) together with KA (Figure 2A) and found no significant differences between KA- and KA+FTY720-treated animals, neither in seizure-like score (Figure 2B) or in neuronal death in CA3 region, as assessed by quantification of Nissl’s stained sections (Figure 2C,D). In a second set of experiments, we used a chronic treatment with FTY720, more similar to the *in vitro* approach. Specifically, in addition to the previously described *icv* injection, rats in the KA+FTY720 experimental group were treated with a prophylactic *ip* injection of FTY720 (1 mg/kg) followed by daily *ip* injections of FTY720 (1 mg/kg) until sacrifice (Figure 3A). Behavioural observation after treatment with KA revealed that FTY720 slightly ameliorated the symptoms (Figure 3B) and reduced neuronal degeneration as well as the extent of the lesion in CA3 hippocampal region (Figure 4A,B). Indeed, quantification of the damaged area showed a significant reduction in KA+FTY720-treated animals as compared to their KA-treated counterparts (Figure 3C,D), indicating a neuroprotective action of FTY720 against KA-induced neurodegeneration *in vivo*. Histological analysis further confirmed the protective effects of FTY720 as the number of FJC^+^ degenerating neurons was lower in the CA3 region of KA+FTY720-treated animals as compared to KA only group (Figure 4C). Moreover, the KA-treated group had significantly lower amount of NeuN^+^ cells in CA3 than KA+FTY720-treated animals (Figure 4D).

### FTY720 reduces activation of microglial cells following KA-induced hippocampal neuronal death

**Figure 5:**
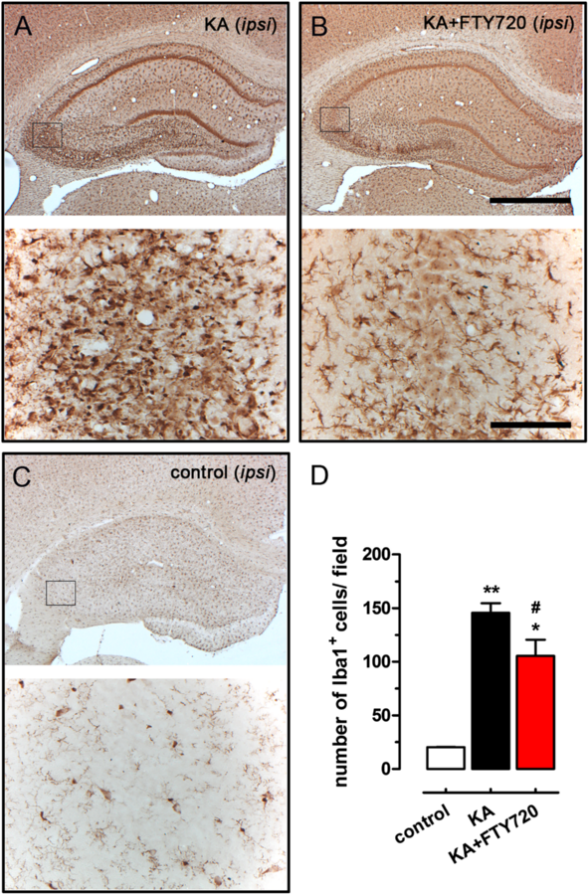
FTY720 reduces microgliosis in CA3 region after *icv* injection of kainic acid (KA). Representative photomicrographs of ipsilateral hippocampal coronal sections of KA-treated **(A)**, KA + FTY720-treated **(B)** and control **(C)** animal groups, immunostained with anti-Iba1 antibody (bright field microscopy). Images on the top of each panel are low magnification photomicrographs showing the entire ipsilateral dorsal hippocampus (scale bar 1 mm). The black frame represents the size field used for counting Iba1^+^ microglial cells in the CA3 region. Images on the bottom of each panel illustrate the area used for microglial cell counting (scale bar 100 μm). **(D)** Quantification of the number of Iba1^+^ microglial cells at CA3 region in control, KA-treated and KA + FTY720-treated animals. Three fields per slice were used to cover the CA3 region and to count the Iba1^+^ microglial cells at the site of injury, and a total of three slices per animal were analysed. Data are expressed as the mean ± SEM of positive cells in ipsilateral CA3 region per slice (control: *n* = 2; KA and KA + FTY720 *n* = 9 from three independent sets of experiments). Student’s *t* test: *P < 0.05 and **P < 0.01 *vs* control; ^#^ *P* < 0.05 *vs* KA.

In order to evaluate of FTY720 on the pro-inflammatory milieu associated with KA-induced seizure-like behaviour and hippocampal degeneration, we focused our attention on the main effectors of neuroinflammation in the brain, namely microglial cells. At 3 days post *icv* of KA, reactive microglia showed large somata and thick primary processes localized in the hippocampus at the site of injury, as revealed by Iba1 immunostaining (Figure 5A). In vehicle-treated rats, microglia were uniformly distributed throughout the hippocampus and presented a less reactive phenotype, with fine and extended processes and small cell bodies (Figure 5C). The number of Iba1^+^ cells in ipsilateral CA3 was counted, and the corresponding graph (Figure 5D) shows a significantly lower number of Iba1^+^ cells in CA3 of the KA + FTY720 group (Figure 5B) than in CA3 of KA group (Figure 5A).

### FTY720 modulates microglia responses *in vitro*

**Figure 6:**
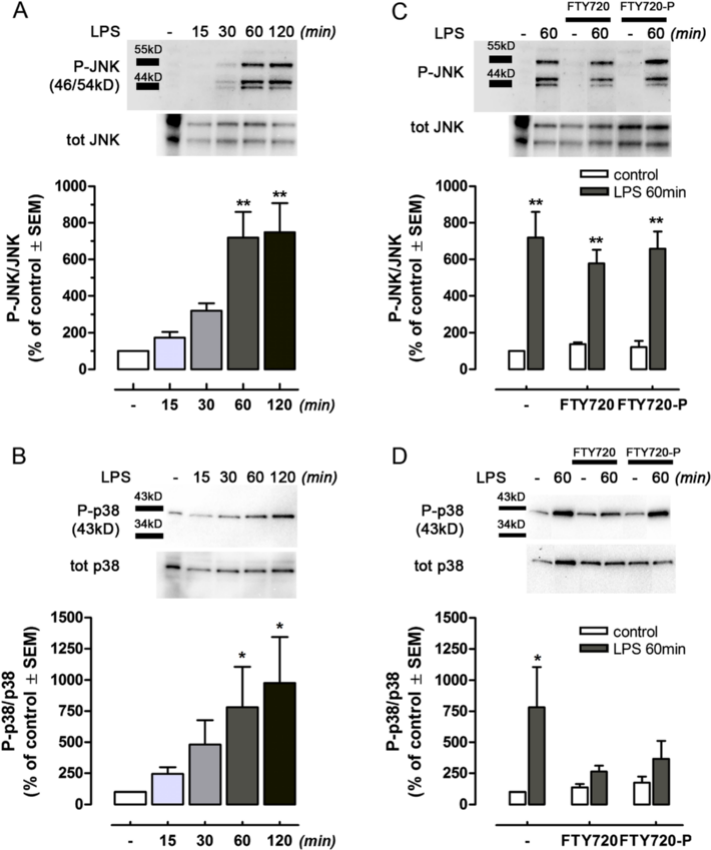
FTY720 modulates inflammatory responses of lipopolysaccharide (LPS)-activated microglia. Microglial cells treated with LPS (100 ng/ml, DMEM without serum) showed time-dependent phosphorylation of JNK **(A)** and p38 **(B)** MAPKs, as determined by Western blot analysis. **(C)** Pre-treatment for 24 h with FTY720 or FY720-P did not reduce LPS-induced phosphorylation of JNK at 60 min, while a significantly effect was shown on LPS-induced p38 activation at the same time point **(D)**. Levels of phosphorylated target protein were normalized to the expression of the corresponding non-phosphorylated form and expressed as percent of untreated cells. The insets on the top of each graph show corresponding, representative Western blots. Lanes have been cropped from the original gel, and minimal adjustments were made to the brightness and contrast settings to improve the clarity of the figure. All lanes shown are from the same gel. Original uncropped blots are shown in Additional file 1: Figure S1. Values are given as mean ± SEM, *n* ≥ 3. Statistical analysis: A and B, one-way ANOVA, *P < 0.05 or **P < 0.01 *vs* untreated cells; C and D, two-tailed *t* test, *P < 0.05 or **P < 0.01 *vs* internal control without LPS.

MAPKs signalling is one of the most important pathways involved in microglia-mediated inflammatory responses to brain injury [44,45]. To elucidate the mechanisms by which FTY720 influence microglia activity, we studied the effects of FTY720 in LPS-induced activation of MAPKs pathways in primary microglia (Figure 6 and Additional file 1: Figure S1). LPS (100 ng/ml) led to activation of JNK and p38 which peaked at around 60 min of stimulation (Figure 6A, B). Pre-treatment of cells with FTY720 or FTY720-P (100nM, similar to what was used previously, [27]) resulted in a significant reduction of LPS-dependent phosphorylation of p38 (Figure 6D), while no changes were observed in the phosphorylation of JNK (Figure 6C), indicating a specific action of FTY720 in modulating microglia responses associated with p38 activation.

## Discussion

In the present study, we describe the neuroprotective action of FTY720, the first oral treatment approved for relapsing-remitting MS, using *in vitro* and *in vivo* models of excitotoxic neuronal death which are relevant to acute CNS injury (for example, cerebral ischemia, traumatic brain injury) and chronic neurodegenerative pathologies (for example, epilepsy, amyotrophic lateral sclerosis, multiple sclerosis, and others) [46].

Despite the emerging role of FTY720 as an effective agent reducing neurodegeneration and promoting reparative mechanisms in different neurological diseases, little is known about the mechanism of action and cellular targets in the brain. Non-immunological, anti-inflammatory actions S1PRs-mediated on glial cells have been associated to the beneficial effects of FTY720 [15,26,47,48]. Conversely, a direct neuroprotective effect on neurons is highly debated, although neurons express S1PRs [12,14,49] and thus may be primarily influenced by FTY720, but the issue is still unclear. Previous studies reported a lack of direct protection of FTY720-P *in vitro*, in cortical primary neurons against glutamate excitotoxicity or oxidative stress-induced cell death [15], or in hippocampal neuronal cell cultures subjected to hypoxia [16]. Conversely, we observed in our *in vitro* models (that is, cortical primary and organotypic cultures) a neuroprotective effect of FTY720 against excitotoxic concentrations of NMDA. Both the *in vitro* models we used for testing the effect of FTY720 exclude the component of the peripheral immune system, thus providing evidence of an action of the drug on neurons, independent of its immunomodulatory function. We chose non-phosphorylated FTY720 relying on previous studies revealing its efficacy in neuronal and oligodendrocyte progenitor cell cultures [30,50]. Moreover, Sphk2 is the predominant S1P-synthesizing isoform expressed in brain tissues [51,52], and a recent work showed how FTY720 was rapidly taken up by human SH-SY5Y neuroblastoma cells and FTY720-P was generated in the nucleus by SphK2 [52], implying that phosphorylation of FTY720 may occur also in our neuronal and organotypic cultures. We showed that FTY720, although exogenously applied in the non-phosphorylated form, reduced neuronal death induced with NMDA, but the effect occurred only in case of prolonged treatment, pre- and post-NMDA. In turn, as shown in Figure 1A, the highest dose of FTY720 (1,000 nM) is toxic in our experimental conditions, an effect that may be due to receptor-independent mechanisms, as previously reported [43].

We obtained similar protective results in cortical organotypic cultures subjected to excitotoxicity. However, in organotypic cultures the higher dose of FTY720 (100 nM) failed to reduce neuronal death, while it still maintained effectiveness on primary cultures. It should be stressed, in this case, the greater complexity of organotypic cultures compared to primary neuronal cultures, the former preserving cytoarchitecture, cellular composition and cell-cell interactions of the source brain region [37,38]. Moreover, organotypic cultures derive from post-natal brains, while neuronal cultures are from embryonic brains, and more mature neurons can present different responses to a same stimulus compared to embryo-derived neurons. Therefore, mechanisms other than those taking place in primary cultures may contribute to or interfere with FTY720-induced neuroprotection, in particular those arising from neuron-glia interactions in organotypic cultures. However, the use of the organotypic culture tool and the effectiveness, albeit weak, of FTY720 also in this experimental model may be very useful for further screening and characterization of protection mechanisms recruited by this drug, without requiring whole animal studies.

Our *in vitro* findings are consistent with recent studies showing a direct, S1P_1_R-mediated, neuroprotective action of FTY720-P [29,30] and non-phosphorylated FTY720 [30] in primary cortical cultures subjected to NMDA excitotoxicity [29]. Pre-treatment appears critical for non-phosphorylated FTY720 efficacy and may partially explain opposite results obtaining in other *in vitro* studies [15]. Thus, Di Menna *et al*. showed that non-phosphorylated FTY720, which presented neuroprotective effects similar to FTY720-P in their experiments, lost its protective activity when applied during or after excitotoxic insult, whereas FTY720-P kept it [30]. Pre-treatment could be necessary to allow intracellular phosphorylation of FTY720 and transport of FTY720-P outside the cells leading to the activation of S1PRs that directly promote neuroprotection or can result in accumulation of other mediators of neuroprotection that in turn would counteract NMDA-induced toxicity. Supporting the last hypothesis, pre-treatment with FTY720-P increases BDNF levels in neurons and mediates neuroprotection in a BDNF-dependent manner [29]. Recent findings suggest a fundamental and more general role of BDNF release in FTY720-mediated neuroprotection. Firstly, FTY720-P-driven up-regulation of neuronal BDNF attenuated oligomeric Aβ-induced neurotoxicity in primary mouse cortical neurons [21]. Secondly, FTY720 restored normal BDNF expression levels in the brain in a mouse model of Rett syndrome [29] and in mice injected with Aβ [23], ameliorating locomotor and cognitive functions, respectively.

Together, our data further support the hypothesis of direct neuroprotective properties of FTY720 in neurons and contribute to clarify the effective conditions against NMDA-mediated excitotoxicity, in terms of concentrations and importance of the pre-treatment.the overall neuroprotective effect of FTY720, in particular when the complexity of the experimental models augments, as in the case of organotypic cultures and *in vivo* model (see Discussion below).

We further assessed *in vivo* the results obtained *in vitro*, using unilateral *icv* injection of KA as a model of excitotoxicity able to induce a pronounced neuronal death in hippocampus, particularly in ipsilateral CA3. Inflammatory processes also contribute to neuronal damage [33,34] allowing to study both the effect of FTY720 on neuronal death and on neuroinflammation. We found that a prophylactic dose followed by daily treatment with FTY720 for 3 days significantly decreased KA-induced hippocampal degeneration. However, protection in these experimental conditions was not achieved with a single dose of FTY720, which is consistent with our *in vitro* assays in cortical neurons as well as with findings in other animal models of degenerating diseases [8,10,19]. In addition, despite the observed reduction of neuronal death with FTY720, seizure-like behaviour improved modestly in line with previous observations [53]. To our knowledge, this is the first demonstration of a neuroprotective action of FTY720 against KA-induced hippocampal neurodegeneration and, together with its beneficial role in a model of lithium/pilocarpine-induced status epilepticus in rats [26], reveals that FTY720 is a potential novel drug in the treatment of epilepsy.

Accumulation of microglia at the site of injury occurs in response to KA-induced neuronal degeneration [33,35] and may contribute to neuronal damage by producing pro-inflammatory cytokines, free radicals and enzymes that further worsen the final outcome of injury [34,35]. Accordingly, we observed a robust increase in the number of microglial cells with an activated morphology at the site of injury 3 days after KA application, which was reduced by FTY720 treatment. These findings are consistent with those obtained in the model of lithium/pilocarpine-induced status epilepticus in rats, where authors found reduced neuronal loss, decreased activation of microglia and astrocytes in the hippocampus and lower expression of interleukin-1 β (IL-1β) and tumour necrosis factor α (TNFα) at 4 days post-status epilepticus following FTY720 treatment [26]. Reduced production of inflammatory mediators by microglia may in turn contribute to the reduction of neuronal death [26]. In addition, astrogliosis also contributes to KA-induced brain injury [31,32,46], and effects of FTY720 on astrocytes have been observed *in vitro* and *in vivo* [26,48,54]. Therefore, astrocytes may also contribute to FTY720-mediated neuroprotection of KA toxicity.

The fact that FTY720 reduces microgliosis and contributes to neuroprotection of KA-induced excitotoxicity prompted us to investigate the mechanisms of FTY720 in modulating microglial responses. Microglia are endowed with S1P receptors, and their expression varies depending on the activation state [12,27,55]. FTY720, via activation of S1P_1_ receptors, down-regulates activated microglial production of pro-inflammatory cytokines TNFα and IL-1β following lysophosphatidyl choline-induced demyelination [28]. Likewise, the production of pro-inflammatory cytokines was lowered by FTY720-P in LPS-activated primary microglial cells, while production of neurotrophic factors BDNF and GDNF increases [27]. However, little is known about modulation by FTY720 of intracellular signalling pathways regulating microglial transcriptional activity and responses. MAPKs are critical intracellular signalling pathways involved in microglial activation and production of cytokines [44,45]. We stimulated microglial cell cultures with the bacterial endotoxin LPS, well known as activator through TLR4 of MAPKs pathway, and an inducer of M1-like, pro-inflammatory and neurotoxic phenotype in microglia [56]. We found that LPS treatment induced a time-dependent activation of JNK and p38 MAPKs which is differentially affected by FTY720, lowering the activation of the later while no significant effects were observed on the JNK pathway. We obtained similar results with FTY720-P suggesting that this form is responsible for reducing activation of p38 signalling pathway.

Albeit previous reports suggested that p38 MAPK may play a critical role in harmful microglial activation in acute brain injury [44], *in vivo* relevance of the differential regulation by FTY720 of p38 and JNK activation on microglial cells needs further elucidation. We propose that FTY720 directly interferes with the neurotoxic microglia activation profile, as demonstrated by the modulation of p38 pathway, and it promotes a neuroprotective phenotype that ultimately acts in concert with a direct neuroprotective action of the drug on neurons. Moreover, FTY720-induced release of neurotrophins *in vivo*, from neurons and/or microglia can act as endogenous neuroprotectants and produce a microenvironment favourable to neuroprotection and neuroregeneration. Taken together, our results provide new evidence showing that FTY720 negatively regulates pro-inflammatory signalling pathways converging into p38 phosphorylation in microglia.

## Conclusions

In summary, we provide evidence of the neuroprotective role of FTY720 against excitotoxic neuronal death, both in cortical neuronal and in organotypic cultures, as well as *in vivo* in KA-induced hippocampal degeneration, whereby FTY720 attenuates microgliosis. Furthermore, we show that FTY720 modulates the microglia inflammatory phenotype *in vitro* by reducing LPS-mediated activation of p38 MAPK signalling pathway. Thus, FTY720 shares both direct neuroprotective and anti-inflammatory properties that can contribute to overall neuroprotection. In particular, the potential of FTY720 to switch microglia phenotype from a detrimental to a protective one represents a therapeutic mechanism for attenuating acute and chronic CNS damage.

## Additional file

Additional file 1: Figure S1. Original Western blots. FTY720 modulates cropped to generate Figure 6. Areas outlined by the red box identify the region which was cropped for generating the insets used in Figure 6 of the manuscript.

## Abbreviations

Aβ: amyloid β
ANOVA: analysis of variance
BBB: blood brain barrier
BDNF: brain-derived nerve factor
BSA: bovine serum albumin
CNS: central nervous system
CTF: corrected total fluorescence
DAB: 3,3′-diaminobenzidine
DMEM: Dulbecco’s modified Eagle’s medium
DMSO: dimethyl sulfoxide hydrochloric acid
EAE: experimental autoimmune encephalomyelitis
FJC: Fluoro-Jade C
FTY720-P: FTY720-phosphate
GDNF: glial-derived nerve factor
HBSS: Hank’s balanced salt solution
*icv*: intracerebroventricular
Iba1: ionized calcium-binding adapter molecule1
IL-1β: interleukin-1 β
*ip*: intraperitoneally
KA: kainic acid
LDH: lactate dehydrogenase
LPS: lipopolysaccharide
MAPKs: mitogen-activated protein kinases
MS: multiple sclerosis
NeuN: neuronal nuclei
PBS: phosphate buffered saline
PDL: poli-D-lysine
PI: propidium iodide
S1P: sphingosine-1-phosphate
S1PRs: S1P receptors
SphK2: sphingosine kinase 2
TBS: tris buffered saline
TNFα: tumour necrosis factor α
