# Supplementary figures and images for: FTY720 attenuates excitotoxicity and neuroinflammation
Source: J Neuroinflammation. 2015 May 8;12:86. doi: 10.1186/s12974-015-0308-6 (PMC4429813; doi:10.1186/s12974-015-0308-6)

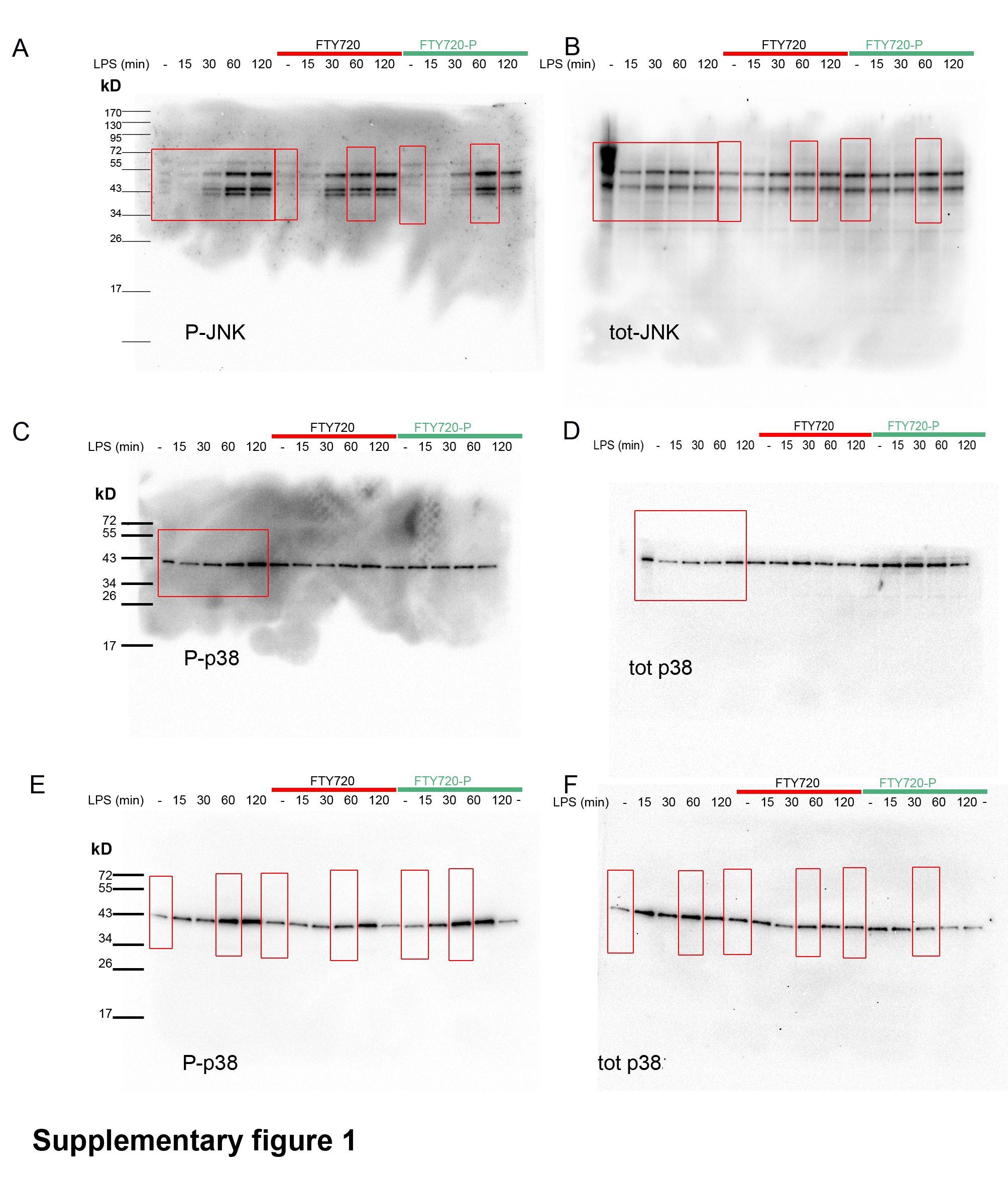

Supplement: Additional file 1: Figure S1. — Original Western blots. FTY720 modulates cropped to generate Figure 6. Areas outlined by the red box identify the region which was cropped for generating the insets used in Figure 6 of the manuscript. [file 12974_2015_308_MOESM1_ESM.tif]
